# Genome mining reveals the prevalence and extensive diversity of toxin–antitoxin systems in *Staphylococcus aureus*

**DOI:** 10.3389/fmicb.2023.1165981

**Published:** 2023-05-24

**Authors:** Jie Xu, Ying Wang, Fang Liu, Guangcai Duan, Haiyan Yang

**Affiliations:** Department of Epidemiology, School of Public Health, Zhengzhou University, Zhengzhou, China

**Keywords:** *Staphylococcus aureus*, toxin-antitoxin systems, phylogroup, genomic, stress

## Abstract

**Introduction:**

*Staphylococcus aureus (S. aureus)* is a highly pathogenic and adaptable Gram-positive bacterium that exhibits persistence in various environments. The toxin-antitoxin (TA) system plays a crucial role in the defense mechanism of bacterial pathogens, allowing them to survive in stressful conditions. While TA systems in clinical pathogens have been extensively studied, there is limited knowledge regarding the diversity and evolutionary complexities of TA systems in *S. aureus*.

**Methods:**

We conducted a comprehensive *in silico* survey using 621 publicly available *S. aureus* isolates. We employed bioinformatic search and prediction tools, including SLING, TADB2.0, and TASmania, to identify TA systems within the genomes of *S. aureus*.

**Results:**

Our analysis revealed a median of seven TA systems per genome, with three type II TA groups (HD, HD_3, and YoeB) being present in over 80% of the strains. Additionally, we observed that TA genes were predominantly encoded in the chromosomal DNA, with some TA systems also found within the Staphylococcal Cassette Chromosomal mec (SCCmec) genomic islands.

**Discussion:**

This study provides a comprehensive overview of the diversity and prevalence of TA systems in *S. aureus*. The findings enhance our understanding of these putative TA genes and their potential implications in *S. aureus* ecology and disease management. Moreover, this knowledge could guide the development of novel antimicrobial strategies.

## 1. Background

*Staphylococcus aureu*s (*S. aureus*) is a prominent hospital- and community-acquired pathogen with a mortality rate ranging from 15 to 60%, posing a critical threat to global public health (Lowy, 1998; Robinson and Enright, 2004; Chuang and Huang, 2015). In addition to being a pathogen, *S. aureus* survives well in different environments, including in water, in air, on the surface of the skin, in animals, and even in frozen food (Xu et al., 2012; Bao et al., 2017). Several studies have reported the induction of viable but non-culturable (VNBC) status accompanied by changes in gene expression profiles upon the exposure of *S. aureus* to environmental stresses such as low temperature, nutrient-limiting conditions, high salt, low pH, and UV-induced conditions (Li et al., 2020; Jiang et al., 2021; Magalhães et al., 2021; Yan et al., 2021). Despite the fact that several stress response pathways have been identified (Feng et al., 2008; Garzoni and Kelley, 2009; Magalhães et al., 2021), it remains poorly understood how *S. aureus* responds to environmental stresses in epiphytic and free-living conditions or maintains virulence genes in the absence of persistent host selection pressure.

Toxin–antitoxin (TA) systems are small genetic modules involved in *S. aureus* persistence (Ma et al., 2019; Jurenas et al., 2022), stress survival (Schuster and Bertram, 2016), and biofilm formation (Schuster and Bertram, 2016; Abd El Rahman et al., 2021). They are usually composed of a two-gene operon encoding a pair of interacting proteins: a stable toxin protein that can harm the host cell and an antitoxin protein that can neutralize homologous toxins in the cell. Additionally, an extra regulator is present in some TA systems. Bacterial TA systems are classified into eight types based on the molecular characteristics of antitoxins and the mode by which they interact with toxins (Ai et al., 2019). Types I, III, and VIII are characterized by small non-coding RNA, whereas other types of TA systems are characterized by toxins and antitoxins that are proteins (Jurenas et al., 2022). The TA system was initially identified in the plasmid of *Escherichia coli* (*E. coli*), and researchers characterized it as a selfish genetic element due to its post-segregational killing effect (Ogura and Hiraga, 1983). Subsequent studies have identified abundant TA systems located on chromosomes and additional functions of TA systems, including phage defense (Ogura and Hiraga, 1983; Bobonis et al., 2022), antibiotic resistance (Pinel-Marie et al., 2014), virulence (Wood and Wood, 2016), and biofilm information (Wood and Wood, 2016; Ma et al., 2019). For example, in *E. coli*, MazEF reportedly degrades total mRNA to mediate programmed cell death, and MqsA specifically regulates the expression of specific genes to regulate biofilm formation. In *Bacillus subtilis* (*B. subtilis*), overexpression of TxpA and YqcG toxins would affect the morphology of the developing biofilm, while the toxin TxpA could lyse and dissolve the pre-established *B. subtilis* biofilm (Bloom-Ackermann et al., 2016). In *Salmonella enterica* serovar Typhimurium, the Hha–TomB TA system was shown to play a significant role in persister cell formation, programmed cell death, and host immune response (Paul et al., 2022). While TA systems have been described for several prevalent clinical pathogens, including *E. coli* (Amitai et al., 2009; Jurenas et al., 2021), *Salmonella* (Huguet et al., 2016; Stårsta et al., 2020; Paul et al., 2022), *Vibrio parahaemolyticus* (Pazhani et al., 2021), and *Klebsiella pneumoniae* (Mataseje et al., 2014; Kang et al., 2020; Narimisa et al., 2020), the diversity and composition of TA systems remain less studied in *S. aureus*. Re-examining all TA modules in *S. aureus* will provide a better understanding of the TA family in *S. aureus*, especially in light of the abundance of reference genomics in public databases.

In this study, we performed *in silico* screening using genome mining techniques on 621 isolates that were sampled from various sources to predict the TA operons. Afterward, the TA operons obtained from the screening step were classified and clustered by setting a strict alignment threshold. The abundance and diversity of TA systems in *S. aureus* were simultaneously studied to characterize the distribution of TA systems in *S. aureus*. This study provides important insights into how these bacteria survive in different hostile environments, and it will help design better strategies to target and develop new treatments for infections. Insights into the details of the VNBC status of *S. aureus* and contributing factors will serve to help develop better treatments for the infection or perhaps better drug targets.

## 2. Materials and methods

### 2.1. Isolate collection

Genome assemblies (FASTA), annotated genomes (GBK and GFF), and protein sequences (FAA) for each corresponding isolate record were retrieved from the esteemed NCBI database (https://www.ncbi.nlm.nih.gov/) between 1 January 2021 and 31 August 2021. The thresholds used for quality metrics included a minimum genome size of 2.5 Mb, a minimum N50 value of 100 kb, and a quality score of at least 90. The relevant literature was meticulously searched, and the esteemed BioSample database (http://www.ebi.ac.uk/biosamples) was utilized to gather contextual information regarding the isolates, including their origin and time of isolation. A custom Python script was used for this purpose and is available at https://github.com/apiaoa123/BiosampleFetcher for repeatability and transparency. To ensure the uniqueness of our dataset, we manually checked each plasmid sequence to ensure that it was not a duplicate entry present in the full *S. aureus* genome entries in either of the databases. We compared the plasmid sequences against the corresponding *S. aureus* chromosome sequences from the same isolate using the basic local alignment search tool (BLAST) and removed any duplicates from our datasets.

### 2.2. Multi-locus sequence typing, methicillin-resistant *S. aureus*, methicillin-susceptible *S. aureus* classification, and staphylococcal cassette chromosomal *mec* typing

Sequence types (STs) and clonal complexes (CCs) of all isolates were determined from whole-genome sequencing (WGS) data and queried against the sequences in the PubMLST database (Jolley et al., 2018) (https://pubmlst.org), which is a public database for molecular typing and microbial genome diversity. To differentiate MRSA and MSSA, we performed BLAST against the *mec* gene in all isolates (e-value < 1e-05, query coverage > 70, and BLAST identity > 90) and classified the strains as MRSA and MSSA based on the presence or absence of homolog strains. In addition, the literature related to strains was searched for auxiliary identification. SCC*mec* typing was allocated by SCC*mec*Finder (IWG-SCC, 2009) (https://cge.cbs.dtu.dk/services/SCCmecFinder/) based on default parameters.

### 2.3. *In silico* screening and mining for TA systems

The toxin–antitoxin system was identified, analyzed, and categorized for whole genomes of *S. aureus* strings using SLING 2.0.1 (Horesh et al., 2018) (https://github.com/ghoresh11/sling), an open-source command line tool based on hidden Markov models (HMM) alignments to annotated TA genes with no limitations. The nucleotide, annotation, and toxin domain databases contained in the program served as the default input to search for the ORFs (run SLING-prepare and scan steps with default parameters). A CDS is considered a hit if its HMMER bit score is at least 20 against the previously characterized and predicted overall sequences/profiles of the toxin CDS contained in the program. Hits with homologous toxin domains were named separately with an e-value cutoff of 0.01 and a previously reported identity score of 30%. We applied the parameters -Mo (maximum overlap between toxin and antitoxin) 20 (bp) and -Md (maximum distance between toxin and antitoxin) 150 (bp) in the filter step and left all other parameters as default (toxin length: 30–200 aa, antitoxin length: 50–150 aa). Following the filtering, we set the -mi (minimum amino acid identity) parameter of the group step to 75 (bp) to cluster the amino acid sequences of the highly conserved toxins and antitoxins. SLING was run on the high-performance computing platform at the National Supercomputing Center in Zhengzhou. To obtain the complete TA output, we also identified TA systems through TADB2.0 (Xie et al., 2018) (https://bioinfo-mml.sjtu.edu.cn/TADB2/), a widely used database for TA identification, and TASmania (Xie et al., 2018) (https://shiny.bioinformatics.unibe.ch/apps/tasmania/), a new database for identifying TA candidates based on guilt-by-association strategies. The TAFinder tool for TADB2.0 was run with the same parameters as SLING, and the input file was the strain comment file (GBK). The TASer tool for TASmania was run with e-values ranging from 0.02 to 1e-100, and the results were filtered using the same parameters.

In addition, the nucleotide files of toxin and antitoxin genes downloaded from the abovementioned database were also aligned by the BLASTN command line tool to identify TA genes in each isolate. Finally, all outputs were merged, and records were filtered by manual retrieval with the following criteria: (1) two genes and both of them were defined as hypothesis proteins; (2) two short genes were organized as an operon encoding toxin and antitoxin proteins; and (3) the distance between the two genes ranges from −20 to 150 bp. TA groups found in more than 80% of isolates of all species were classified as “prevalent”, more than 20% of isolates were classified as “common”, and the rest were classified as “rare”.

### 2.4. Housekeeping gene searching and phylogenetic analysis

To explore the phylogenetic distribution of the TA systems in *S. aureus*, we crafted a meticulously crafted phylogenetic tree utilizing the whole-genome multi-locus sequence typing (wgMLST) approach for population analysis. The allele-calling process for *S. aureus* involved utilizing the polished genome assemblies and the chewBBACA v3.0.0 software (Silva et al., 2018), utilizing the wgMLST 1850-loci Pasteur schema obtained from the esteemed Chewie-NS website “https://chewbbaca.online” (downloaded on 14 April 2023). Subsequently, the wgMLST clustering analysis was carried out using ReporTree v.1.0.1 “https://github.com/insapathogenomics/ReporTree” (Mixão et al., 2022) with GrapeTree employing the MSTreeV2 method and default parameters. TA groups were shown in outer circles as a heatmap based on the annotated gene number in each isolate. Both tree and TA groups were visualized using the Interactive Tree of Life online website (Letunic and Bork, 2021) (iTOL: https://itol.embl.de/#).

### 2.5. GC content calculation, antimicrobial resistance genes, and virulence gene identification

The GC content of the putative TA gene was extracted to characterize the signs of TA systems obtained by horizontal transfer. We compared the GC content of TA genes with the GC content of the whole genome of *S. aureus*. AMR genes were identified by ABRIcate (https://github.com/tseemann/abricate) with CARD (Alcock et al., 2020), ResFinder (Bortolaia et al., 2020), and AMRFinder (Feldgarden et al., 2019) databases. Virulence genes were detected using ABRIcate with the VFBD database (Chen et al., 2005).

### 2.6. Statistical analyses

The correlation between the *S. aureus* genome size and TA counts was analyzed using the Pearson correlation test, and the results were considered significant if the *p*-value was < 0.05. It was considered that the Pearson correlation coefficient value |r| above 0.7 is relatively strong; |r| between 0.4 and 0.7 is considered moderate, and those below 0.4 is considered weak. Given the non-normality of the data, the Mann–Whitney U-test and the Kruskal–Wallis H-test were used to compare the differences between TA counts in different isolate origins and types of strains. As geographic, host origin, and typing data were discrete and unordered, the chi-squared test was used to determine whether or not the presence of the TA system was independent of strain contextual information. Fisher's exact test was used to determine whether or not there is a significant association between a given TA system and a given AMR gene. When testing the association between a given TA with multiple AMR genes (or virulence genes), the false discovery rate (FDR) correction was used to correct the *p*-values. All the above statistical analyses were performed using NumPy (Harris et al., 2020) and SciPy (Virtanen et al., 2020) modules of Python.

## 3. Results

### 3.1. Diversity of *S. aureus* in the database

**Figure 1:**
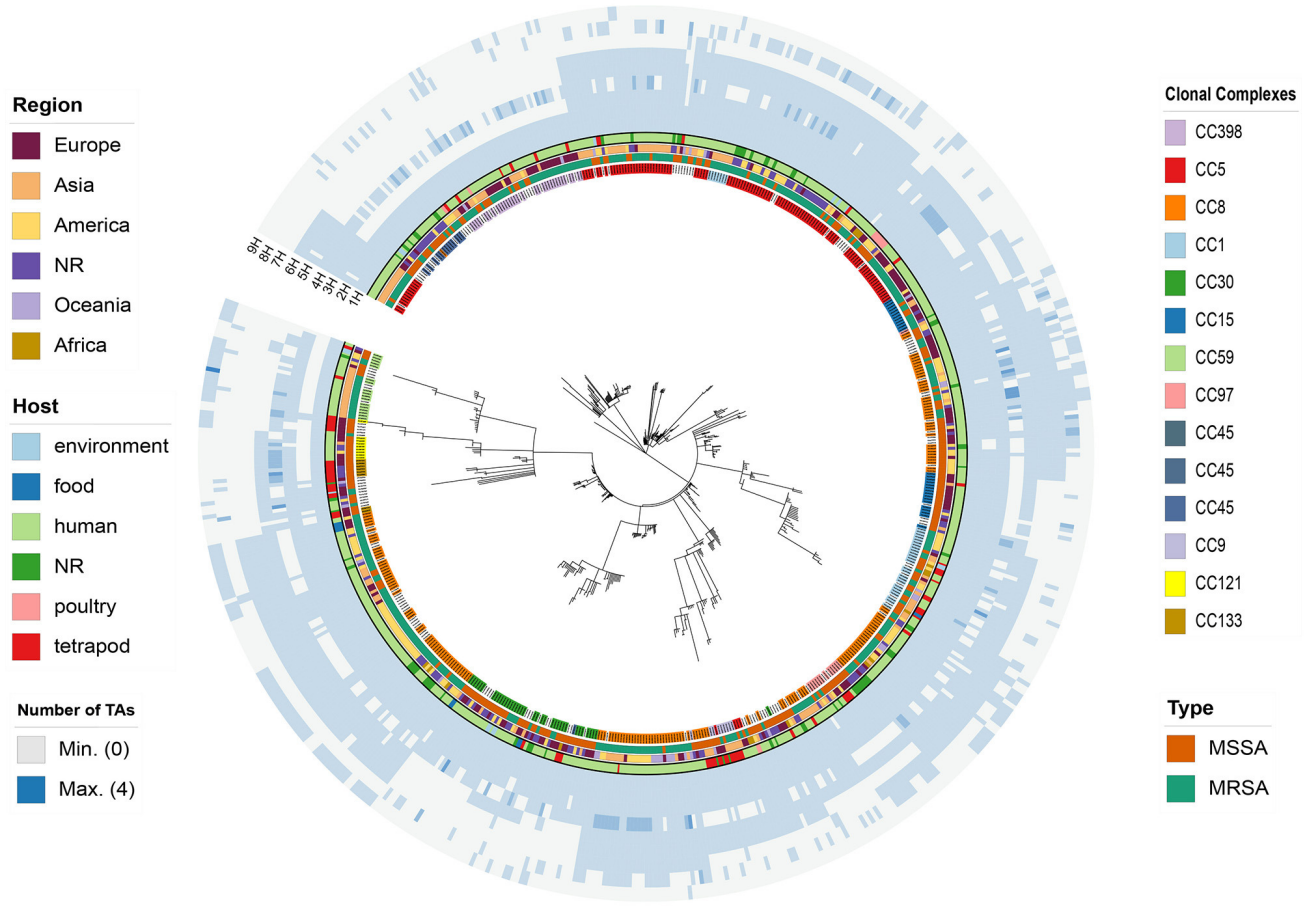
wgMLST-based phylogenetic tree of 621 *S. aureus* strains is depicted with colored rings, each representing different characteristics. Starting from the innermost ring and moving outward, the rings indicate the clonal complexes (CCs), type, region of isolation, source of isolation, 1H-HD, 2H-HD_3, 3H-YoeB_toxin, 4H-ANT-AntA, 5H-YoeB_toxin-ParE_toxin, 6H-AHSA1, 7H-Peptidase_M78-DUF955, and 8H-Bro-N-ANT, respectively.

A total of 621 *S. aureus* whole-genome sequences, including 621 chromosome sequences and 502 plasmid sequences, were obtained from the NCBI database (Supplementary Table 1), resulting in a total of 1123 entries. Among these, information on the geographical origin was available for 494 strains (79.55%, Figure 1). The majority of the strains (163, 26.25%, Figure 1) were isolated from Europe, followed by the Americas with 149 strains (24.0%), Asia with 143 strains (23.03%), Oceania with 31 strains (5%), and Africa with 17 strains (2.73%). A comprehensive record of 560 (90.18%) strains was available with regard to their host origin. Of these, the majority were derived from human tissues, making up a substantial 76.97% of the total. Strains originating from livestock accounted for 9.18%, while a mere nine strains were traced to poultry origin, amounting to 1.45%. A small fraction of the strains, specifically 10 in total, were linked to an environmental origin (constituting 1.61%), and a mere six strains were traced back to food origin, amounting to only 0.97%. Furthermore, the time of collection of the strains with known isolation dates ranged from 1,884 to 2,021, encompassing the oldest isolates in our collection that were obtained in 1884 from pleural fluid of a German (NCBI BioSample ID: SAMN06480665). In total, 507 strains had a known time of isolation, while the time of isolation for 114 strains remained unknown.

### 3.2. *S. aureus* genomes encoded abundant putative toxin–antitoxin systems

A total of 3, 960 TA systems were obtained using SLING, including 44 TA groups and 106 antitoxin groups (Supplementary Table 2). Instead of following a single database to find TA genes, we searched for other databases/tools in the literature to validate putative TA genes and found TADB2.0 and TASmania. TADB2.0 provides information for both TA loci and genetic characteristics. TASmania is the newest discovery-oriented database available for free. We performed extensive screening using TADB2.0 and TASmania as references and applied the same parameters as SLING. A total of 2, 005 TA pairs hits were obtained from TADB2.0 (Supplementary Table 3), and 1, 015 hits were obtained from TASmania (Supplementary Table 4). Among the three tools employed for TA gene identification, namely TADB2.0, TASmania, and SLING, it was observed that SLING exhibited superior performance, identifying a notably higher number of putative TA genes. Notably, 27.5% of the experimentally validated TA genes were encompassed in the output of SLING, bestowing invaluable insights into the identification and characterization of TA systems, as well as their distribution in *S. aureus*. Hence, we chose to base our subsequent analysis on the output of SLING, considering its incorporation of experimentally validated TA genes and its potential for more comprehensive identification of TA loci in our study. As an initial step in unraveling the roles of TA systems in the ecology of *S. aureus*, we classified TA groups based on their frequency in strains. Among them, only three TA groups were found to be highly prevalent, being present in over 80% of the isolates (Figure 1, Supplementary Table 5). These included 1H with the HD domain, which was detected in 619 out of 621 genomes (99.68%, Supplementary Table 5), 2H with the HD_3 domain, present in 570 out of 621 genomes (91.79%, Supplementary Table 5), and 3H with the YoeB domain, present in 542 out of 621 genomes (87.28%, Supplementary Table 5). HD is a phosphoryl hydrolase that may be the domain of the toxin in TADB, but its exact function is unknown. YoeB is ribosome-dependent mRNA interferon that inhibits translation initiation in the same manner as YoeB-ec in *E. coli* (Yoshizumi et al., 2009), and it has been proven to be involved in antibiotic resistance and biofilm formation in *S. aureus* (Cherny and Gazit, 2004; Schuster and Bertram, 2016). There were six TA groups that were (4–9H, Supplementary Table 5) classified as the “common” group and the remaining 35 groups were classified as the “rare” group (10–44H, Supplementary Table 5).

### 3.3. TA system abundance was associated with genome size, plasmid presence or absence, GC content, and MRSA or MSSA status

The toxin–antitoxin systems are associated with the stability of mobile genetic elements (MGEs), with which they can be horizontally transferred. Because genome sizes increase due to the integration of MGEs or the presence of plasmids, we hypothesized that a large genome would have a greater number of TA systems. The genome size of *S. aureus* included in this study ranged from 2.66 to 3.13 Mb, with a mean of 2.85 Mb (Supplementary Table 1). TA system counts varied from two to 11 within strains, with a median of seven and a mode of seven. Spearman correlation test showed that the genome size presented a significant moderate correlation with TA system counts (r = 0.53, *P* < 0.0001, Supplementary Figure 1). In addition, the counts of TA systems in genomes with plasmids significantly exceeded those in genomes without plasmids (Mann–Whitney U-test, *P* < 0.001, Table 1). Only 14 (0.35%) TA systems were found in 13 (2.59%) plasmids, and the majority (3, 946/3, 960, 99.65%) of TA systems are encoded by 621 (100%) chromosomes (Supplementary Table 2). Of those, 18H-PemK and 43H-PemK were identified only in plasmids, while the more prevalent eight groups in *S. aureus* (prevalent: 1H-HD, 2H-HD_3, 3H-YoeB; common: 4H-Ant-AntA, 5H-YoeB-ParE, 6H-AHSA1, 7H-Peptidase_M78-DUF955, and 8H-Bro-N-ANT) were exclusively identified in the chromosome.

**Table 1:** Correlation of TA counts and characteristic variables of *S. aureus*.

	**Statistic**	***p*** **-value**	**Method**
Type	−0.661	0.509	z-test in Possion regression
Plasmid present or not	33384.5	0.003^*^	Mann-Whitney U test
Size	−4.55	1.71 x 10^−12^^*^	z-test in Possion regression
Region	49.53	1.73 x 10^−9^^*^	Kruskal-Wallis H test
ST	155.17	1.06 x 10^−31^^*^	Kruskal-Wallis H test
CC	163.52	1.76 x 10^−33^^*^	Kruskal-Wallis H test
Host	17.58	0.004^*^	Kruskal-Wallis H test

Given the non-normality of the data, the Mann–Whitney U-test and the Kruskal–Wallis H-test were used to compare the differences between TA counts in different isolate origins and types of strains. As MRSA and MSSA status might be involved with strain size, a Poisson regression was used to explore the association between TA counts and type. The Statistic column indicates the statistical value corresponding to the statistical method.

* The differences were statistically significant.

The GC content of toxin hits was extracted and compared with the GC content of the genomic sequence to identify the signal of TA systems acquisition by horizontal transfer. Our results showed that the GC content of all three toxin genomes in the prevalent group was significantly different from the whole-genome sequence, and five of the six TA groups in the common group differed significantly from the whole-genome sequence (Supplementary Figure 2).

Considering that the TA system in *S. aureus* is associated with β-lactam resistance and that the number of TA systems in the genome has a superimposed effect on its function, we compared the abundance of the TA system in MRSA and MSSA. There were 354 (57%) MRSA strains and 267 (43%, Supplementary Table 1) MSSA strains among the 621 strains, of which 2 strains carried *mecC* genes and 352 (56.69%) strains carried *mecA* genes. The difference between the abundance of TA loci in the MRSA and MSSA genomes was statistically significant, and the TA system was more abundant in MRSA than in MSSA (*P* < 0.001, Supplementary Figure 1). Given that MRSA strains exhibit a higher abundance of TA genes compared to MSSA and possess larger genomes, we conducted Poisson regression analysis to examine the relationship between the number of TAs and genome size as well as type. The results indicated that the number of TAs was positively correlated with size (*P* < 0.001, Table 1), but the type was not associated with TA number (*P* = 0.509, Table 1).

In contrast to MSSA, MRSA carries a *mec* operon that encodes a different penicillin-binding protein, PBP2a, which exhibits low methicillin affinity and makes it insensitive to all β-lactam antibiotics. The resistance determinant *mecA* and *mecC* are located in the SCC*mec* element, which also harbors several other resistance genes, and this element accelerates the spread of resistance genes. Therefore, we hypothesized that the difference in TA abundance in MSSA and MRSA is attributable to SCC*mec*. To validate our hypothesis, we annotated the TA system of SCC*mec* genomic islands and evaluated the diversity of the TA system in SCC*mec*. Of the 354 MRSA strains included, 348 strains were able to be classified by SCC*mec* typing, and nine types and seven subtypes were detected. Using a detailed sequence search, we identified only 22 toxin hits across seven SCC*mec* genomic islands (three type IV, two type V, one type III, and one type VIII), including 11 groups (Table 2).

**Table 2:** TA groups in SCC*mec* genomic island.

**Assembly**	**SCC*mec***	**SCC*mec*_start**	**SCC*mec*_end**	**Toxin**	**T_start**	**T_end**
GCA_001515665.1	SCC*mec*_type_III(3A)	40872	89860	42H_NTP_transf_2	47875	48174
GCA_001580495.1	SCC*mec*_type_IVc(2B)	1192002	2048547	3H_YoeB_toxin	1648003	1648269
GCA_001580495.1	SCC*mec*_type_IVc(2B)	1192002	2048547	4H_ANT, AntA	1249997	1250749
GCA_001580495.1	SCC*mec*_type_IVc(2B)	1192002	2048547	5H_ParE_toxin, YoeB_toxin	1704556	1704822
GCA_001580495.1	SCC*mec*_type_IVc(2B)	1192002	2048547	10H_HD	1335603	1336268
GCA_003193665.1	SCC*mec*_type_V(5C2&5)	1737666	2591826	3H_YoeB_toxin	2136549	2136815
GCA_003193665.1	SCC*mec*_type_V(5C2&5)	1737666	2591826	5H_ParE_toxin, YoeB_toxin	2079994	2080260
GCA_003193665.1	SCC*mec*_type_V(5C2&5)	1737666	2591826	10H_HD	2448661	2449326
GCA_003193665.1	SCC*mec*_type_V(5C2&5)	1737666	2591826	12H_PemK_toxin	2530338	2530877
GCA_004153365.1	SCC*mec*_type_IVc(2B)	1543836	2420613	3H_YoeB_toxin	1947665	1947931
GCA_004153365.1	SCC*mec*_type_IVc(2B)	1543836	2420613	5H_ParE_toxin, YoeB_toxin	1889717	1889983
GCA_004153365.1	SCC*mec*_type_IVc(2B)	1543836	2420613	10H_HD	2262264	2262929
GCA_005706855.1	SCC*mec*_type_IVa(2B)	68795	2800448	1H_HD	1634420	1635004
GCA_005706855.1	SCC*mec*_type_IVa(2B)	68795	2800448	2H_HD_3	791191	791859
GCA_005706855.1	SCC*mec*_type_IVa(2B)	68795	2800448	3H_YoeB_toxin	2430660	2430926
GCA_005706855.1	SCC*mec*_type_IVa(2B)	68795	2800448	4H_ANT, AntA	1531452	1532204
GCA_005706855.1	SCC*mec*_type_IVa(2B)	68795	2800448	5H_ParE_toxin, YoeB_toxin	2487997	2488263
GCA_005706855.1	SCC*mec*_type_IVa(2B)	68795	2800448	7H_DUF955, Peptidase_M78	1857678	1858163
GCA_005706855.1	SCC*mec*_type_IVa(2B)	68795	2800448	8H_ANT, Bro-N	2038297	2039085
GCA_005706855.1	SCC*mec*_type_IVa(2B)	68795	2800448	10H_HD	2118726	2119391
GCA_014844155.1	SCC*mec*_type_V(5C2)	2271402	2717322	10H_HD	2558493	2559158
GCA_900474525.1	SCC*mec*_type_VIII(4A)	38165	79897	17H_GNAT_acetyltran	68744	69241

### 3.4. TA abundance was related to the host origin, regional origin, and clonal complexes

Some studies have previously reported that TA systems are more prevalent in isolates that survive in unfavorable environments and that some TA systems also have regional and host distribution differences. We, therefore, assessed whether the origin of strain isolation (host origin and regional origin) was associated with TA system abundance. The abundance of TA systems varied significantly among strains from different hosts and regions, according to the Kruskal–Wallis H-test (*P* = 0.004 and *P* < 0.001, Table 1). Strains from a given host or a given region often have a given clonal complex, and MLST clonal complexes provide valuable insights into the population genetics of *S. aureus*. To understand the determinants affecting the abundance of TA systems, we compared the abundance of TA systems between different CCs. Statistically significant differences were found between strains with different ST types regarding the number of TA systems (*P* < 0.001, Table 1). ST398 had significantly more TA than several other groups, and there were noticeable correlations between ST398 and 1H, 2H, and 4H than other groups (Supplementary Figure 3A). More specifically, all 32 ST398 isolates harbored the 1H-HD gene and the 2H-HD_3 gene, and 23 out of 32 ST398 isolates harbored at least one 4H-ANT-AntA gene.

Furthermore, to elucidate and compare the coding prowess of TA systems across diverse lineages of *S. aureus* and unveil their phylogenetic characteristics, we generated a population analysis phylogenetic tree using a wgMLST-based approach. Subsequently, we mapped the abundance information of toxin genes onto the phylogenetic tree for comprehensive analysis and interpretation (Figure 1). Different CCs exhibited disparate toxin-encoding capabilities, as evidenced by the absence of 2H in CC59, CC133, and CC121, while 7H was clustered in CC8. To further explore the possible role of type II TA systems in the proliferation of clonal groups of *S. aureus*, the distribution of type II TA systems in different clonal groups of *S. aureus* was analyzed in this study. Among the 621 *S. aureus* strains, 482 strains could be classified into 17 CCs (Table 3), of which the most abundant was CC8 (161/482, 33.40%; Table 3), followed by CC5 (114/482, 23.65%; Table 3). Correlation analysis revealed significant associations, with 7H and 9H being notably linked with CC8 and 1H, 2H, and 4H being considerably associated with CC398. Furthermore, 6H was significantly associated with CC5 compared to other complexes, as depicted in Supplementary Figure 3B. These findings collectively demonstrated that the type of TA systems is correlated with CC typing, and horizontal acquisition of TA genes may play a pivotal role in the evolutionary dynamics of *S. aureus* transmission.

**Table 3:** Clonal complexes of the included isolates.

**CC**	**MRSA**	**MSSA**	**Africa**	**America**	**Asia**	**Europe**	**Oceania**	**Environment**	**Food**	**Human**	**Poultry**	**Tetrapod**
CC1	20 (62.5%)	12 (37.5%)	2 (6.25%)	10 (31.25%)	14 (43.75%)	2 (6.25%)	3 (9.38%)	1 (3.12%)	1 (3.12%)	23 (71.88%)	0 (0.0%)	6 (18.75%)
CC15	9 (32.14%)	19 (67.86%)	0 (0.0%)	2 (7.14%)	3 (10.71%)	19 (67.86%)	1 (3.57%)	0 (0.0%)	0 (0.0%)	26 (92.86%)	0 (0.0%)	1 (3.57%)
CC30	8 (21.62%)	29 (78.38%)	1 (2.7%)	6 (16.22%)	2 (5.41%)	15 (40.54%)	0 (0.0%)	2 (5.41%)	1 (2.7%)	30 (81.08%)	0 (0.0%)	2 (5.41%)
CC398	28 (87.5%)	4 (12.5%)	0 (0.0%)	2 (6.25%)	4 (12.5%)	25 (78.12%)	1 (3.12%)	0 (0.0%)	0 (0.0%)	28 (87.5%)	1 (3.12%)	3 (9.38%)
CC5	89 (78.07%)	25 (21.93%)	0 (0.0%)	25 (21.93%)	36 (31.58%)	25 (21.93%)	2 (1.75%)	3 (2.63%)	0 (0.0%)	91 (79.82%)	5 (4.39%)	2 (1.75%)
CC8	117 (72.67%)	44 (27.33%)	5 (3.11%)	63 (39.13%)	27 (16.77%)	26 (16.15%)	17 (10.56%)	0 (0.0%)	3 (1.86%)	130 (80.75%)	1 (0.62%)	5 (3.11%)

### 3.5. TA system was associated with antimicrobial resistance genes and virulence genes

**Figure 2:**
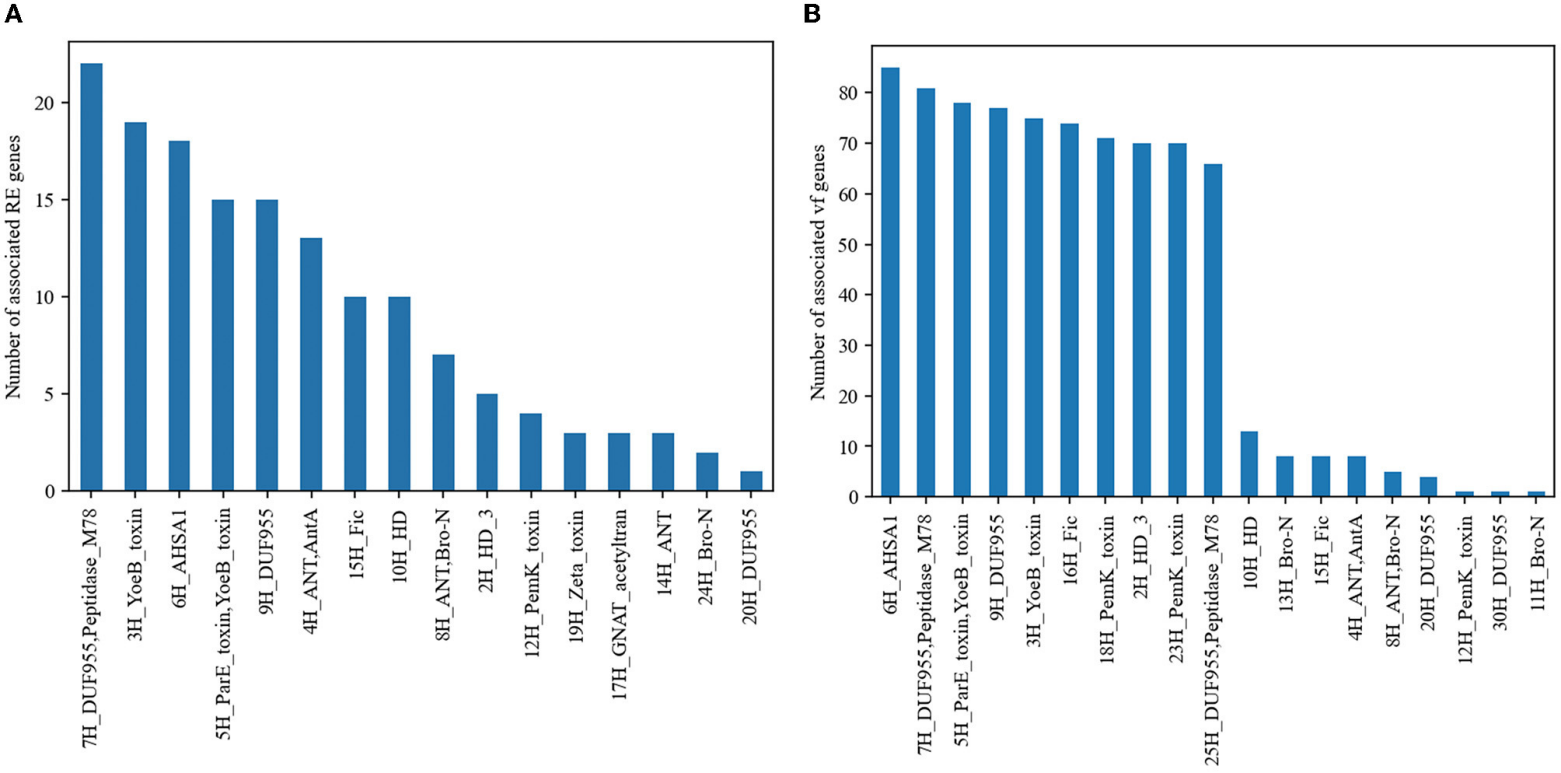
TA systems associated with AMR genes and virulence genes. **(A)** TA groups associated with AMR genes and **(B)** TA groups associated with virulence genes. Only the groups with statistically significant associations are shown in the figure.

Given that the TA system is usually linked to the stability of mobile genetic elements carrying resistance and virulence genes, we searched for physical linkage or co-occurrence between TA systems and marker genes associated with AMR or virulence. Some TA groups are associated with AMR or virulence genes (Figures 2A, B, Fisher's exact test, *P* < 0.05, FDR corrected). Concretely, 16 out of 44 TA groups were significantly associated with AMR genes, and 11 of them were associated with *mecA* genes, which encode for penicillin-binding protein PBP2a that appear on the same contig. Five TA groups with experimentally validated domains, 5H (ParE), 3H (YoeB), 18H (PemK), 23H (PemK), and 12H (PemK) were significantly associated with multiple virulence genes (Fisher's exact test *P* < 0.05, FDR corrected; Figure 2B).

## 4. Discussion

In this study, we performed *in silico* TA identification on genomic public sequencing data and characterized the diversity of TA systems in *S. aureus*. Our analyses of large-scale genomic data demonstrated that the TA systems are prevalent and present in a variety of patterns in *S. aureus*. Among the 621 strains, we found 3, 960 TA systems, which could be classified into 44 groups with strict sequence alignment thresholds. All identified TA systems were type II TA systems, in which the antitoxin typically contains an N-terminal DNA-binding domain for transcriptional self-regulation and a C-terminal domain that binds directly to the toxin counterpart. We categorized the 44 TA groups into three categories (see the “Method” section) based on their frequency in our datasets. Three groups were assigned to “prevalent,” present in over 80% of the strains. Six TA groups were delivered to “common” in over 20% of isolates. The remaining 35 TA groups were classified as “rare.” The most prevalent HD domain is a phosphohydrolase which is possibly involved in signaling, and its exact role remains unclear (Huynh et al., 2015). The group 3H with the YoeB domain is a 50S ribosomal-dependent mRNA cleavage at the A site, which is well-described for its function in biofilm formation and methicillin resistance (Yoshizumi et al., 2009). As 3H with YoeB is widely present in *S. aureus* and associated with biofilm and resistance formation in *S. aureus*, we proposed the hypothesis that YoeB could be a novel antibacterial drug. Another toxin well described in *S. aureus* is a toxin with the Pemk domain, which is thought to be associated with antibiotic tolerance, biofilm formation, growth inhibition, and chronic infection of *S. aureus* (Ma et al., 2019; Abd El Rahman et al., 2021). The other TA groups predicted in this study remain to be validated, and their function in other bacteria is presented in Supplementary Table 6.

A significant linear correlation was found between genome size and TA system abundance, which could be attributed to the fact that larger genomes are associated with a higher presence of MGEs such as plasmids and genomic islands (Casjens, 1998). Consistently, we found a more abundant number of TA systems in genomes with plasmids than in genomes without plasmids. MRSA with SCC*mec* genomic islands encode more TA systems than MSSA with genomic islands without SCC*mec*. We identified the TA system in both plasmid and SCC*mec*. Moreover, in most toxins, the GC content was different from the genome-wide GC content (Almpanis et al., 2018). These results demonstrated that most of the TA system in *S. aureus* is probably derived via horizontal transfer and that SCC*mec* may influence the distribution of TA genes in MRSA.

Our findings revealed notable variations in the abundance of TA systems among strains from different isolate sources. To elucidate the underlying reasons for this divergence, we conducted an in-depth analysis of the distribution of TA systems among various CCs. Given that isolates within the same clonal complex are closely related and that the ST type can provide valuable information in identifying bacterial relationships, we employed these approaches in parallel to gain comprehensive insights into the observed patterns (Robinson and Enright, 2004). For instance, ST9 predominates in most Asian countries, and ST398 is host-specific and prevalent in European countries and North America (Chuang and Huang, 2015). Consistently, the number of toxin genes did differ significantly between the different CCs. A significant correlation was shown between 1H, 2H, 4H, and ST398 concerning the other complexes. In keeping with their ecological uniqueness, strains belonging to the same complexes also share similar phenotypic traits (Feng et al., 2008). In addition, it has been proven to be valuable for identifying the ST of clonal complex clusters relevant to specific ecological niches, with the potential to contribute to the characterization of the transmission pathways of human infections (Robinson and Enright, 2004). ST398 was previously associated with livestock transport, but in recent years, livestock-independent *S. aureus* ST398 has emerged, representing a potential health risk to humans (Zhao et al., 2020). Studies on the association of ST398 with the TA module would provide possible insights into the development of antibacterial drugs. To our knowledge, this is the first study on the association of TA modules with CCs in *S. aureus*, and further experiments are warranted to validate our results.

In this study, we solely identified and characterized TA systems in *S. aureus* at the sequence level without experimental validation or thorough exploration of the functions of these TA systems. Nevertheless, numerous uncertainties remain unresolved, and additional experimental investigation is imperative to substantiate our conclusions. Moreover, although we identified the TA system in the SCC*mec* genomic island, the function of the TA system in SCC*mec* is unknown and more studies are needed to explore. We also acknowledged that there might be limitations associated with the SLING-based approach, and this should be considered in the interpretation of our results. Further validation and comparison with other methods would be valuable to better understand the strengths and weaknesses of each tool. Future studies could also explore the impact of varying stringency thresholds on TA system identification and assess their implications on statistical associations with isolated populations.

In conclusion, we revealed that *S. aureus* encodes multiple TA systems that target different cellular functions upon activation. Based on the abundance pattern and ecological evidence of the role of our results, we hypothesized that the TA system might be an essential evolutionary engine for maintaining the virulence of genes and antimicrobial resistance. Further study of the experimental properties and ecological role of the predicted TA system could provide important insights into the epidemiology and management of diseases caused by *S. aureus*.

## Data availability statement

The datasets presented in this study can be found in online repositories. The names of the repository/repositories and accession number(s) can be found in the article/Supplementary material.

## Author contributions

HY, GD, and JX: conceptualization and writing manuscript drafts. JX, YW, and FL: data acquisition and analysis. HY, GD, and FL: supervision and funding acquisition. All authors: revision of drafts. All authors contributed to the article and approved the submitted version.
