# Supplementary material for: Genome mining reveals the prevalence and extensive diversity of toxin–antitoxin systems in Staphylococcus aureus
Source: Front Microbiol. 2023 May 24;14:1165981. doi: 10.3389/fmicb.2023.1165981 (PMC10244574; doi:10.3389/fmicb.2023.1165981)
Supplement: Supplementary file 2 [file Data_Sheet_1.DOCX]

***Supplementary Material***

# Supplementary Tables

**Supplementary** **Table 1** Basic information of the included strains.

**Supplementary Table 2** Output of SLING.

**Supplementary Table 3** Output of TADB2.0.

**Supplementary Table 4** Output of TASmania.

**Supplementary Table 5** Identified toxin-antitoxin group

**Supplementary Table 6** Toxin-antitoxin systems predicted in *S. aureus*, their toxin function, and which biological processes they involve.

# Supplementary Figures


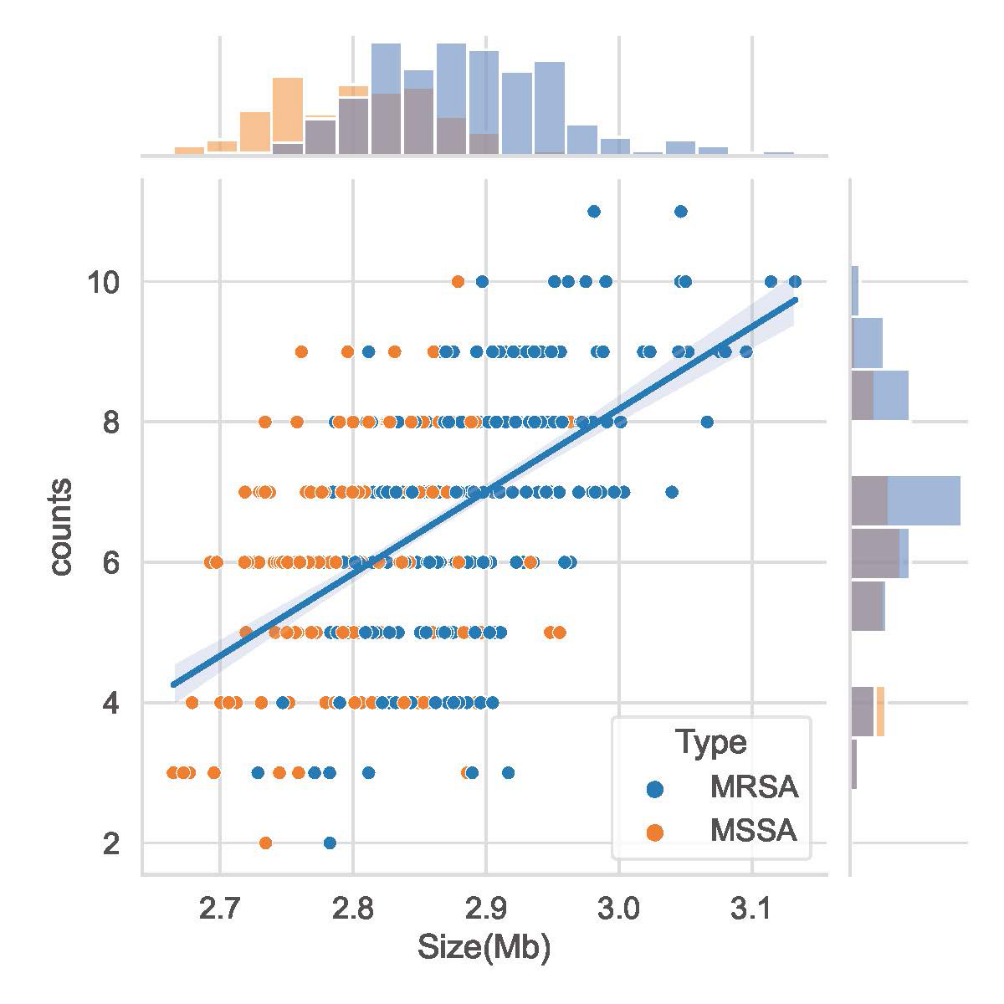


**Supplementary Figure 1** Relationship between genome size and the number of potential TAs systems in *S. aureus*. Histogram corresponds to the horizontal and vertical coordinates, respectively, and the types of *S. aureus* are marked with different colors as Methicillin-resistant *S. aureus* (MRSA) and Methicillin-susceptible *S. aureus* (MSSA), in which the MRSA genome size is significantly larger than that of MSSA.


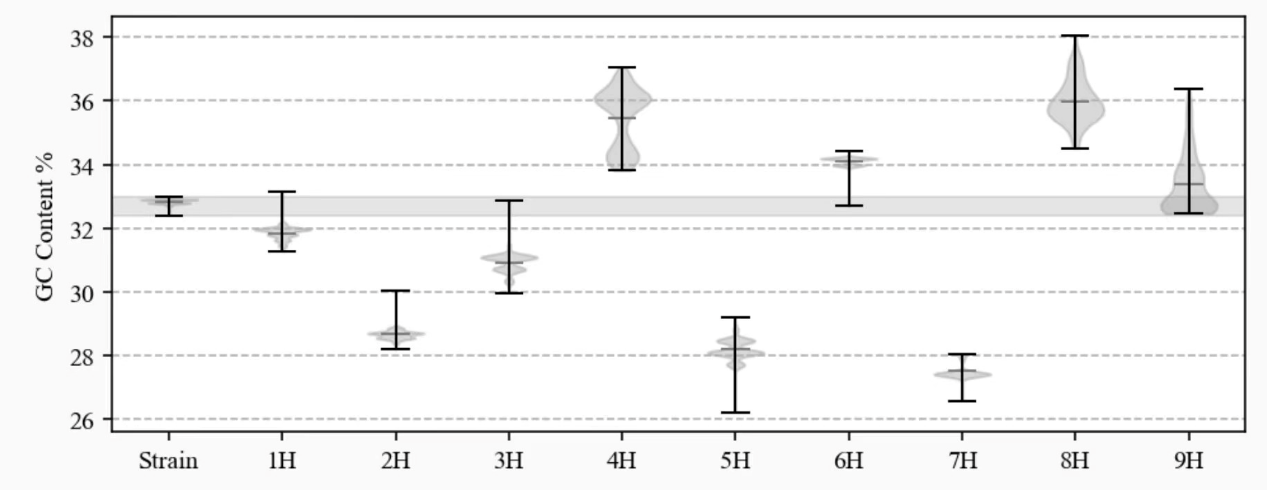


**Supplementary Figure 2** Variation in GC content of each TA system. 621 genomic GC contents of the genomes and the first nine toxin hits of the identified strains, shaded areas indicate the GC content of the genomes.


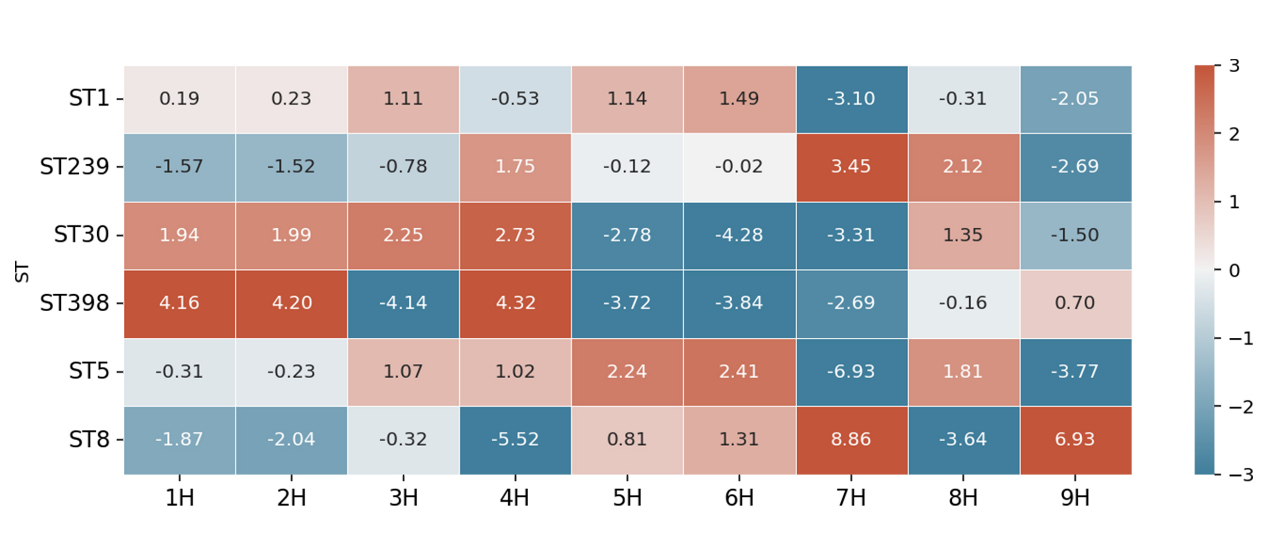


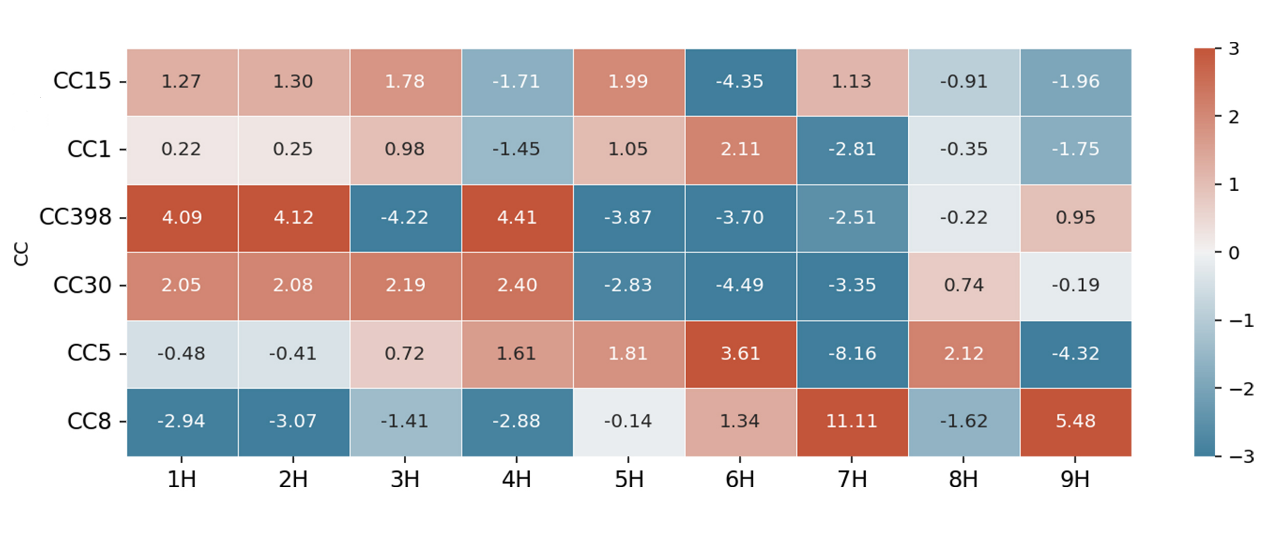


**Supplementary Figure 3** Heatmap showing the distribution of TA group across clonal complexes. **(A)** Association analysis between different strains of ST type and different groups of TA systems; **(B)** Association analysis between different strains of clonal complexes and different groups of TA systems. The heatmap color response the probability of occurrence of the given TA systems in the related species. The standard residuals (z-score) of the chi-square test are marked on the corresponding grid points to indicate whether the distribution of TA system is correlated with different attributes. If the standard residual is higher than 1.96 (or lower than -1.96), the probability of occurrence of a given TAs is significantly (*P* < 0.05) higher (or lower) than that of the other strains for that attribute.
